# Mussel-inspired resilient hydrogels with strong skin adhesion and high-sensitivity for wearable device

**DOI:** 10.1186/s40580-024-00419-4

**Published:** 2024-03-21

**Authors:** Stalin Kondaveeti, Geonjun Choi, Sarath Chandra Veerla, Somi Kim, Jaeil Kim, Hee Jin Lee, Unnikrishnan Kuzhiumparambil, Peter J. Ralph, Junyeob Yeo, Hoon Eui Jeong

**Affiliations:** 1 Department of Mechanical Engineering, Ulsan National Institute of Science and Technology (UNIST), Ulsan, 44919 Republic of Korea; 2 Department of Physics, Kyungpook National University, 80 Daehak-Ro, Bukgu, Daegu, 41566 Republic of Korea; 3 Climate Change Cluster, University of Technology Sydney, Ultimo, NSW Australia

**Keywords:** Conductive hydrogel, Self-adhesion, Self-healing, Mussel adhesion, Wearable sensors

## Abstract

Stretchable and self-adhesive conductive hydrogels hold significant importance across a wide spectrum of applications, including human–machine interfaces, wearable devices, and soft robotics. However, integrating multiple properties, such as high stretchability, strong interfacial adhesion, self-healing capability, and sensitivity, into a single material poses significant technical challenges. Herein, we present a multifunctional conductive hydrogel based on poly(acrylic acid) (PAA), dopamine-functionalized pectin (PT-DA), polydopamine-coated reduction graphene oxide (rGO-PDA), and Fe^3+^ as an ionic cross-linker. This hydrogel exhibits a combination of high stretchability (2000%), rapid self-healing (~ 94% recovery in 5 s), and robust self-adhesion to various substrates. Notably, the hydrogel demonstrates a remarkable skin adhesion strength of 85 kPa, surpassing previous skin adhesive hydrogels. Furthermore, incorporating rGO within the hydrogel network creates electric pathways, ensuring excellent conductivity (0.56 S m^–1^). Consequently, these conductive hydrogels exhibit strain-sensing properties with a significant increase in gauge factor (GF) of 14.6, covering an extensive detection range of ~ 1000%, fast response (198 ms) and exceptional cycle stability. These multifunctional hydrogels can be seamlessly integrated into motion detection sensors capable of distinguishing between various strong or subtle movements of the human body.

**Supplementary Information:**

The online version contains supplementary material available at 10.1186/s40580-024-00419-4.

## Introduction

In recent years, flexible wearable devices have undergone significant advancements, driven by their distinctive attributes such as flexibility, adaptability, high sensitivity, and seamless interfacing capabilities with target surfaces. These attributes have paved the way for innovative applications, including electronic skin [1, 2], human–machine interfaces [3, 4], biomedical devices [5–7], and soft robotics [8, 9]. The development of these flexible wearable devices primarily hinges upon soft materials, encompassing elastomers, hydrogels, and various polymers. Hydrogels, particularly, have emerged as a critical material due to their biomaterials-like composition, exceptional biocompatibility, and the capacity to tailor mechanical and chemical properties finely [10, 11]. However, traditional hydrogels exhibit inferior mechanical properties compared to biological materials [12]. Accordingly, recent research efforts have predominantly focused on enhancing the mechanical properties of hydrogels by modulating their crosslinking networks by incorporating various chemical substances [13]. Simultaneously, integrating self-healing properties into a range of hydrogel materials has been an active avenue of exploration to address the limitations of conventional hydrogels. Additionally, researchers have actively investigated the incorporation of various conductive fillers such as carbon nanomaterials (CNTs) [14], conductive polymers [15, 16], inorganic salts [17], and liquid metals [18–21] to confer electrical conductivity upon hydrogels, thereby expanding their applicability in wearable and flexible devices.

Despite significant progress in developing advanced hydrogels with improved mechanical and electrical properties, challenges remain in achieving reliable self-adhesion, especially when adhering to surfaces such as human skin. The inherent properties of skin, such as surface roughness, wrinkles, hair, and sweat, pose a major obstacle to achieving seamless and intimate contact formation. In addition, dynamic movements of the skin, such as bending, can easily cause hydrogel adhesives to detach from the surface. As a result, many existing hydrogel-based wearable devices require external adhesive tapes or bandages for secure connection to the skin or other substrates [22, 23]. This partial contact often results in elevated interfacial impedance, diminished sensitivity, and causing instability in the detection of human physiological signals, thereby significantly compromising device performance. The adhesion strength of many existing hydrogel-based wearable devices typically falls within the range of 5–30 kPa [24, 25].

To address these challenges, researchers have pursued a mussel-inspired chemistry approach to fabricate self-adhesive hydrogel sensors. By introducing catechol-containing compounds, including dopamine (DA) [26, 27], tannic acid (TA) [28, 29], modified lignin [30], and catechol-modified polysaccharides [31, 32], the innate capability of catechol groups to form robust covalent and non-covalent bonds with polar substrates has been harnessed. These bonds impart unique self-adhesive properties to the hydrogels. However, the self-adhesion properties may gradually weaken due to oxidation of the catechol group, potentially hindering long-term utility. Furthermore, mussel-inspired hydrogels face challenges in providing sufficient dynamic interaction points within the bulk material and at interfaces, complicating efforts to enhance mechanical stretchability, self-healing, and self-adhesion simultaneously. In our previous work, we reported using polyphenols (specifically tannic acid) as adhesive factors to create ionic hydrogels with stable adhesion to skin and solid materials [28]. However, these ionic hydrogels exhibited poor mechanical properties along with low sensitivity [guage factor (GF) = 2.5]. Despite recent advances in hydrogel technology, developing hydrogel-based wearable devices that simultaneously possess strong self-adhesion, robust stretchability, rapid self-healing, and high strain sensitivity remains a significant challenge and requires further exploration.

Herein, we present a highly stretchable, self-adhesive, self-healing, and conductive hydrogel with a high strain sensitivity. This multifunctional hydrogel was developed by incorporating poly(acrylic acid) (PAA), dopamine-functionalized pectin (PT-DA), and polydopamine-coated reduced graphene oxide (rGO-PDA) into a flexible thin film configuration. Our design leverages multiple dynamic interactions, including hydrogen bonds and coordination bonds. These mussel-inspired dynamic interactions within the hydrogels confer remarkable attributes, including exceptional stretchability (2000%), and rapid self-healing at room temperature through physical contact alone (72% recovery in 2 s and 94% recovery in 5 s). In addition, the hydrogel demonstrates remarkable self-adhesion to various substrates with varying chemical compositions and surface roughness. Its outstanding skin adhesion strength of 85 kPa is particularly notable, surpassing that of many existing hydrogel adhesives. Incorporating rGO within the hydrogel network establishes efficient electric pathways, ensuring robust conductivity (0.56 S m^–1^). Consequently, these conductive hydrogels-based mechanical sensors exhibit strain-sensing properties with a remarkable gauge factor (GF) of 14.6, covering an extensive detection range of ~ 1000% and rapid response (198 ms).

## Experimental section

### Materials

Pectin from citrus peels (PT, galacturonic acid, ≥ 74%), dopamine hydrochloride (DA, > 98%), acrylic acid (AA, > 99%), ammonium persulfate (APS, > 98%), 1-ethyl-3-(3-(dimethylamino) propyl) carbodiimide hydrochloride (EDC, > 98%), *N*-hydroxysuccinimide (NHS, > 98%), *N*,*N*′-Methylenebis-(acrylamide) (MBAA, > 98%), Ferric nitrate (Fe(NO_3_)_3_·9H_2_O) and natural graphite oxide (GO) were purchased from Sigma Aldrich, USA. All other chemicals were purchased commercially and used without further purification.

### Synthesis of dopamine-functionalized pectin conjugates (PT-DA)

To prepare the PT-DA, DA was chemically conjugated to the carboxyl group of pectin using EDC and NHS as previously described [33]. In brief, 500 mg of pectin was dissolved in 50 mL of 0.1 M MES buffer with a pH of 6.0. EDC (960 mg) and NHS (576 mg) were slowly added to the pectin solution at a ratio of 1:4:4 (pectin:EDC:NHS), and the mixture was reacted for 1 h to activate the carboxylic group of pectin. Subsequently, DA (948 mg) was added to the solution at a 4:1 molar ratio to pectin, and the reaction was carried out at room temperature for 12 h with vigorous stirring. To eliminate any unreacted DA and purify the final product, the solution was dialyzed (molecular weight cutoff = 8000 Da) against deionized water for 2 days. The resulting mixture was then lyophilized and stored at − 20 °C before use. Structural confirmation of the PT-DA conjugates was performed using ^1^H NMR analysis.

### Preparation of rGO-PDA and PPGP (PT-DA/PAA/rGO-PDA) hydrogels

Initially, GO powder was introduced into a tris buffer solution (10 mM, pH 8.5) to create a 1.0 mg mL^−1^ aqueous dispersion after 30 min of ultrasonication. Subsequently, dopamine was slowly added to the GO dispersion, resulting in a final concentration of 0.5 mg mL^−1^. Vigorous agitation was maintained for 18 h at 25 °C. Dopamine, in this process, acted as a reducing agent, converting GO into rGO. rGO exhibits hydrophobic properties, improved electrical conductivity, and excellent mechanical characteristics compared to GO. The dispersion color changed from brown to black due to the self-polymerization of dopamine onto the GO materials. The GO-PDA dispersion was then washed with deionized water multiple times until the supernatant became colorless and dried at 60 °C for 12 h. The resulting composite material was designated as rGO-PDA [34].

The PT-DA/PAA/rGO-PDA conductive hydrogels were created through in-situ polymerization of acrylic acid (AA) within the PT-DA polymer networks, facilitated by Fe^3+^ as an ionic cross-linker. Initially, PT-DA (2 g) and AA (10 g) were dissolved in 90 mL of deionized water under continuous stirring for 1 h. Following that, rGO-PDA (0.05 g), *N*,*N*′-methylenebis(acrylamide) (MBAA, 0.01 g), and Fe(NO_3_)_3_·9H_2_O (0.025 g) was added to the mixture of PT-DA and AA, and the stirring continued for 2 h. Subsequently, ammonium persulfate (APS, 0.05 g) was added to the mixture for 1 min. Finally, the uniform solution was poured into a mold, which consisted of a pair of glass plates and a silicone spacer with a thickness of 3 mm. The mixture was polymerized at 40 °C for 1 h to obtain the PT-DA/PAA/rGO-PDA hydrogels. The composition details of these hydrogels are provided in Additional file 1: Table S1. For comparison, PAA and PT-DA/PAA hydrogels were prepared under the same conditions.

### Characterization of hydrogels

High-resolution scanning electron microscopy (HR-SEM) images of the conductive hydrogels were obtained using a Hitachi S-4800 scanning electron microscope (Hitachi S-4800). Before imaging, each hydrogel sample was frozen at 0 °C for 24 h and then freeze-dried (SCIENTZ-10N) at − 20 °C for 24 h. A small amount of freeze-dried hydrogel was placed on a jig or sample holder with conductive adhesive. The samples were subsequently coated with a 5 nm thick Pt layer using metal sputtering (K575X sputter coater, Quorum Emitech, UK) to prevent charging. Infrared spectra of the conductive hydrogels were recorded using Fourier-transform infrared (FTIR) spectroscopy (Nicolet iS10, Thermo Fisher Scientific, USA) in the range of 400–4000 cm^–1^, employing the potassium bromide (KBr) tablet method. X-ray diffraction (XRD; Rigaku Ultima(III) analysis was conducted on both GO and rGO samples using an instrument with CuK α radiation (λ = 0.154 nm). The 2θ range was set to 5–60° with a scan speed of 2°/min and a step rate of 0.02 per second. To characterize the chemical composition of GO and rGO-PDA samples, X-ray photoelectron spectroscopy (XPS) analysis was performed using a photoelectron spectrometer (Thermo Scientific, USA).

### Assessment of mechanical characteristics

We conducted mechanical property assessments on the hydrogels using a standard tensile testing machine (Instron 5982, Instron Corporation, USA). To prepare test specimens, we cut them into elongated strips measuring 10 mm in length, 8 mm in width, and 2 mm in thickness. These strips were subjected to tensile testing at a consistent stretching rate of 100 mm/min. We calculated the toughness of the hydrogels by integrating the area beneath the stress–strain curves. Additionally, we determined the elastic moduli (E) by fitting the stress–strain curves during the initial phase of linearity. To assess the compressibility of the hydrogels, we prepared cylindrical samples with a diameter of 20 mm. Compression tests were performed at a strain level of 90%, with a controlled speed of 20 mm/min. The experiments were conducted with a minimum of five samples for each test.

### Adhesion testing

The adhesive properties of the hydrogels were assessed using a specially designed adhesion tester, which was equipped with various substrates, including porcine skin, aluminum, glass, and polystyrene substrates. The normal adhesion strength was determined through the pull-off testing method [35, 36]. The hydrogel samples, with a contact area of 1 cm × 1 cm, were positioned on the lower substrate, and the samples were brought into contact with the upper substrate with a preload of 20 kPa for 10 s. Subsequently, the maximum adhesion strength was measured as the upper substrate was pulled upwards at a speed of 2 mm s^−1^. Each set of samples underwent testing a minimum of five times.

### Evaluation of self-healing properties

To evaluate the mechanical properties after self-healing, we divided hydrogel specimens into two equal halves and brought the resulting segments into contact for varying durations. Following this, the regenerated hydrogel was subjected to tensile tests under the same conditions as the original sample. Additionally, the electrical self-healing capacity of the hydrogel was examined by measuring the recovery of its conductivity. The conductive hydrogels were connected to source measurement equipment (DAQ970A, KEYSIGHT, USA), and the alterations in the hydrogel’s resistance were measured throughout repeated cutting-healing cycles. A light-emitting diode (LED) was integrated into the circuit to improve visualization of electrical properties.

### Evaluation of electromechanical properties of hydrogels

We examined the electrical behavior of the hydrogels using a two-probe method with source-measurement equipment (DAQ970A, KEYSIGHT, USA). The electrical conductivity of the hydrogels, featuring different rGO-PDA contents (ranging from 0.025 to 0.1 g), was assessed by sandwiching them between two copper plates (contact area: 2 cm × 1 cm) with a 1 cm gap. The electrical conductivity (σ) was calculated using the formula: σ = L/(R × S), where L, R, and S represent the length (cm), resistance (Ω), and sectional area (cm^2^) of the test hydrogels, respectively. To evaluate the strain-sensing capabilities of the conductive hydrogels, we measured electrical resistance (R) while applying tensile stress using custom-built strain-controllable equipment. Two opposite sides of the rectangular samples (initial length: 0.5 cm; thickness: 0.2 cm; width: 2 cm) were connected to copper wires, and a constant strain ranging from 0 to 2000% was applied. As the tensile machine stretched the hydrogel, an electrochemical workstation recorded real-time changes in the hydrogel’s resistance. The relative resistance change (ΔR/R_0_) was determined by dividing the resistance change (ΔR = R − R_0_) by the initial resistance, R_0_. To detect human body movements and subtle vibrations, we initially cut the hydrogels into properly sized strips. Subsequently, two wires were connected to both ends of each hydrogel strip. The hydrogels were then directly attached to various parts of the human body.

## Results and discussion

### Design and mechanism of conductive hydrogels

**Fig. 1:**
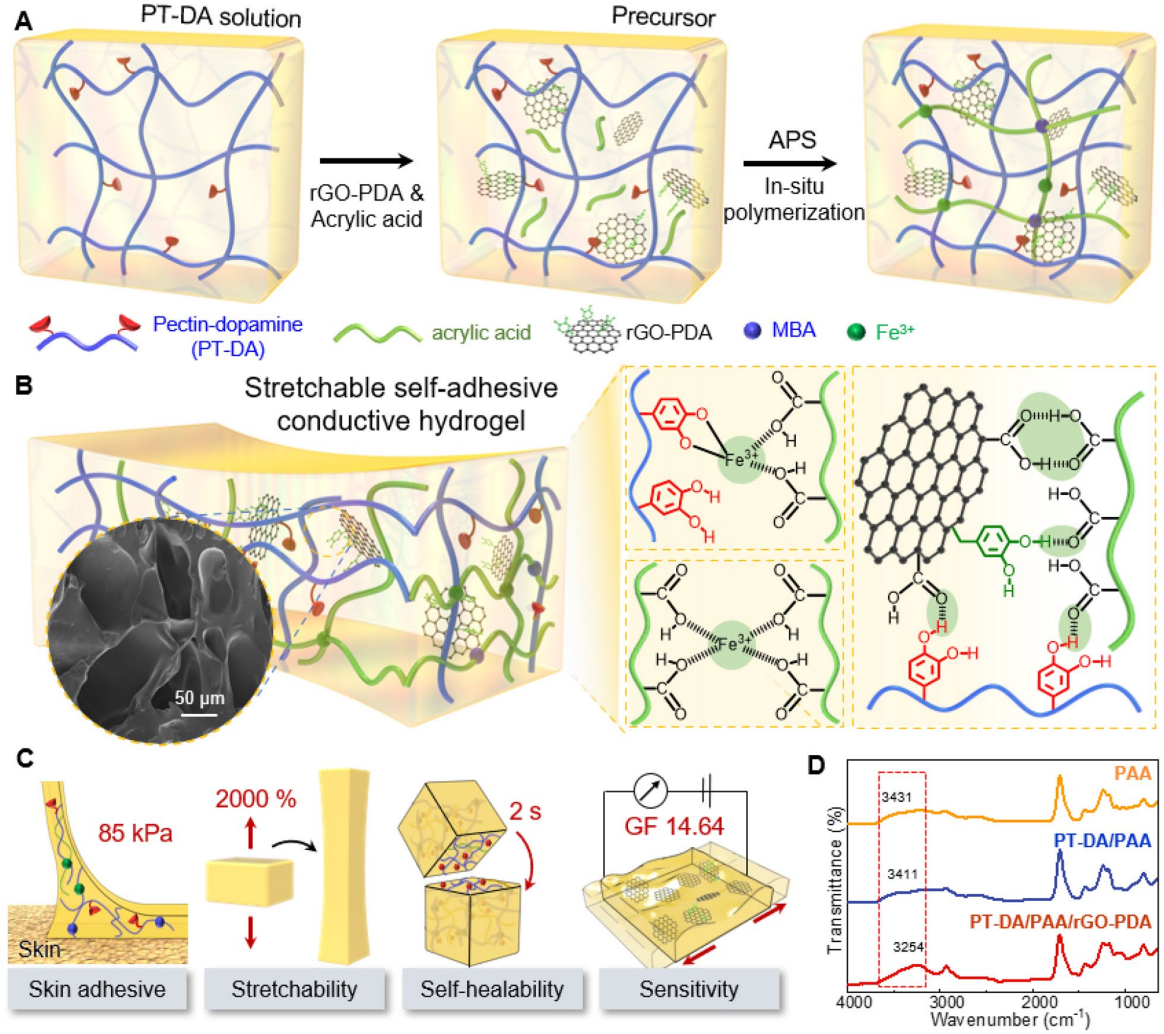
Schematic illustration of multifunctional PT-DA/PAA/rGO-PDA hydrogels. **A** Schematic depicting the synthetic process and chemical structure of multifunctional hydrogels composed of PT-DA, PAA, and rGO-PDA. **B** Possible chemical interactions between PAA, PT-DA, and rGO-PDA within the hydrogel networks. **C** The synthesized hydrogel’s multifunctionality shows strong self-adhesion, high stretchability, room-temperature self-healing, and high strain sensitivity. **D** FTIR spectra of the PAA, PT-DA/PAA, and PT-DA/PAA/rGO-PDA hydrogels

Figure 1 conceptualizes the multifunctional hydrogel with strong self-adhesion, high stretchability, rapid self-healing, and high strain sensitivity. The hydrogel was synthesized through a straightforward one-pot in-situ free radical polymerization process based on dynamic covalent and non-covalent chemistry (Fig. 1A, B). To begin, PT-DA conjugates were synthesized by modifying pectin with dopamine (DA) using the EDC/NHS coupling reaction (Additional file 1: Fig. S1) [33]. The successful modification of DA was verified using ^1^H NMR and FTIR (Additional file 1: Fig. S1). In the ^1^H NMR spectrum, proton peaks in the range of 6.6 to 6.8 ppm are associated with aromatic protons, and the peaks around 2.8 ppm are characteristic of methylene (–CH_2_–) groups (Additional file 1: Fig. S1B) [31]. Similarly, there is a broad absorption peak at 1749 cm^−1^ in the FTIR spectrum of PT-DA, which corresponds to the C=O stretching vibration of the carboxyl group in the pectin polymer (Additional file 1: Fig. S1C). These results demonstrate the successful reaction between dopamine primary amines and carboxyl groups in pectin, leading to the subsequent synthesis of PT-DA (Additional file 1: Fig. S1) [37].

Next, a PDA solution converted graphene oxide (GO) to reduced GO (rGO). PDA was then coated onto the surface of rGO, reducing the surface functional carbonyl and hydroxyl groups of GO to form rGO-PDA (Fig. 1) [34]. The XRD pattern of GO and rGO-PDA is shown in Additional file 1: Fig. S2. The GO peak at 11.5° corresponds to the crystal plane of (001) with a d-spacing of 0.39 nm (Additional file 1: Fig. S2A). The transition from GO to rGO, facilitated by dopamine as a reducing agent, is evident from the broad peak at 26.96°. This peak is recognized as the characteristic peak of rGO indexed as the (002) plane, with a d-spacing of 0.182 nm (JCPDS No # 75-1621) (Additional file 1: Fig. S2B) [38]. The XPS analysis of GO and rGO/PDA is presented in Additional file 1: Fig. S3. In the XPS spectra, characteristic peaks at 288.33 eV (O–C=O), 286.8 eV (O–C–O), and 288.76 eV (C=O) were observed in rGO-PDA (Additional file 1: Fig. S3B), and new peaks at 285.07 eV (C–N) and 290.4 eV (π–π*) indicated the presence of catechol groups in the rGO [39]. Therefore, both XRD and XPS analyses confirm the successful conversion of GO to rGO.

The multifunctional hydrogels were synthesized through a free radical polymerization process, utilizing AA as the monomer, APS as the initiator, and Fe(NO_3_)_3_ as the cross-linking agent. This synthesis occurred in the presence of PT-DA and rGO-PDA. During the hydrogel polymerization, AA was converted into PAA with the assistance of the crosslinker MBAA. Simultaneously, PT-DA played a pivotal role by interacting with PAA through rGO-PDA, acting as a mediator and physically entangling to form the primary network structure of the multifunctional hydrogel. Within the multifunctional hydrogel, covalent and non-covalent bonds constituted the primary types of interactions in the networks, contributing to the hydrogel’s multifunctionality. Chemically crosslinked PAA chains and PT-DA polymers sustained a permanent dense skeleton, efficiently dissipating energy upon external load application. Furthermore, catechol and hydroxyl groups in PT-DA and carboxyl groups in PAA formed extensive inter- and intra-chain hydrogen bonds, enhancing the hydrogel’s high stretchability and elasticity (Fig. 1B). Additionally, non-covalent ionic interactions between Fe^3+^ ions and the carboxylic groups of PAA, as well as catechol groups of PT-DA, formed a reversibly loose network, enabling the hydrogel’s self-healing properties (Fig. 1B).

Thanks to the unoxidized catechol groups of PT-DA, the multifunctional hydrogels (PT-DA/PAA/rGO-PDA; referred to as PPGP hydrogels hereafter) exhibited self-adhesiveness to various substrates. Furthermore, the functionalization groups introduced on GO by dopamine formed strong inner or intermolecular hydrogen bonds, further stabilizing the conductive hydrogel (Fig. 1B). Moreover, rGO-PDA was well-dispersed in the network through hydrogen bonds between rGO-PDA chains and carboxyl groups in PAA, as well as hydroxyl groups in PT-DA chains. As shown in Fig. 1C, the fabricated hydrogels exhibited multifunctional properties: (i) strong adhesion to skin was provided by the free catechol groups on PT-DA and rGO-PDA chains, along with the carboxyl groups from PAA; (ii) metal coordination bonds between Fe^3+^ ions, the carboxylic groups of PAA, and catechol groups of PT-DA imparted excellent self-healing properties to the hydrogels; (iii) the interwoven porous structure of rGO-PDA formed an electronic pathway, resulting in high electrical conductivity and strain sensitivity of the hydrogel; (iv) the toughness and stretchability of the multifunctional hydrogels were achieved through the presence of PAA and pectin.

The interactions and chemical structure formation of PAA, PT-DA/PAA, and PT-DA/PAA/rGO-PDA hydrogel were analyzed using FTIR spectroscopy (Fig. 1D). The characteristic peak for the OH stretching vibration observed at 3431 cm^−1^ represents the intermolecular hydrogen bonds in the PAA chains [40]. Upon the introduction of PT-DA into the PAA hydrogel, a right-shifted peak appeared at 3411 cm^–1^, indicating the formation of stronger hydrogen bonds between PT-DA and PAA. Subsequently, the addition of rGO-PDA resulted in a further shift of the peak to approximately 3254 cm^–1^, implying the formation of enhanced hydrogen bonds between the components. This evidence confirms the successful formation of the multifunctional PPGP hydrogels. Additional file 1: Fig. S4 shows the morphologies of the PAA, PT-DA/PAA, and PPGP hydrogels. The PAA hydrogel displayed smooth surfaces, whereas the PT-DA/PAA and PPGP hydrogels exhibited interconnected irregular porous and loose structures, indicating strong interactions between rGO-PDA, PT-DA, and PAA. These porous structured multifunctional hydrogels efficiently trap conductive carbon from rGO, providing excellent electrical conductivity and strain sensitivity to the hydrogel [41, 42].

### Mechanical behaviors of multifunctional hydrogels

Excellent mechanical properties are crucial for the reliable utilization of hydrogels in various wearable and soft devices, which often undergo repeated and significant mechanical deformations. To investigate the effect of PT-DA and rGO-PDA content on the mechanical properties of multifunctional hydrogels, we prepared hydrogel samples with varying concentrations of PT-DA and rGO-PDA. Initially, we examined the stress–strain behavior of PT-DA/PAA hydrogels with different PT-DA concentrations ranging from 0 to 2.5 wt% (Additional file 1: Fig. S5). The introduction of PT-DA into the PAA hydrogel notably increased both the fracture strength and fracture strain. The fracture strength of the PT-DA/PAA hydrogel exhibited a positive correlation with the PT-DA content, peaking at 70 kPa with 2 wt% PT-DA. This enhancement can be attributed to increased hydrogen bonds within the multifunctional hydrogel networks formed between PT-DA and PAA chains. However, a further rise in PT-DA content to 2.5 wt% resulted in a decrease in fracture strength. This reduction is attributable to the excessive PT-DA content, which can weaken the synergistic effect of hydrogen bonding interactions, ultimately leading to a decreased tensile strength of the PT-DA/PAA hydrogel [43].

**Fig. 2:**
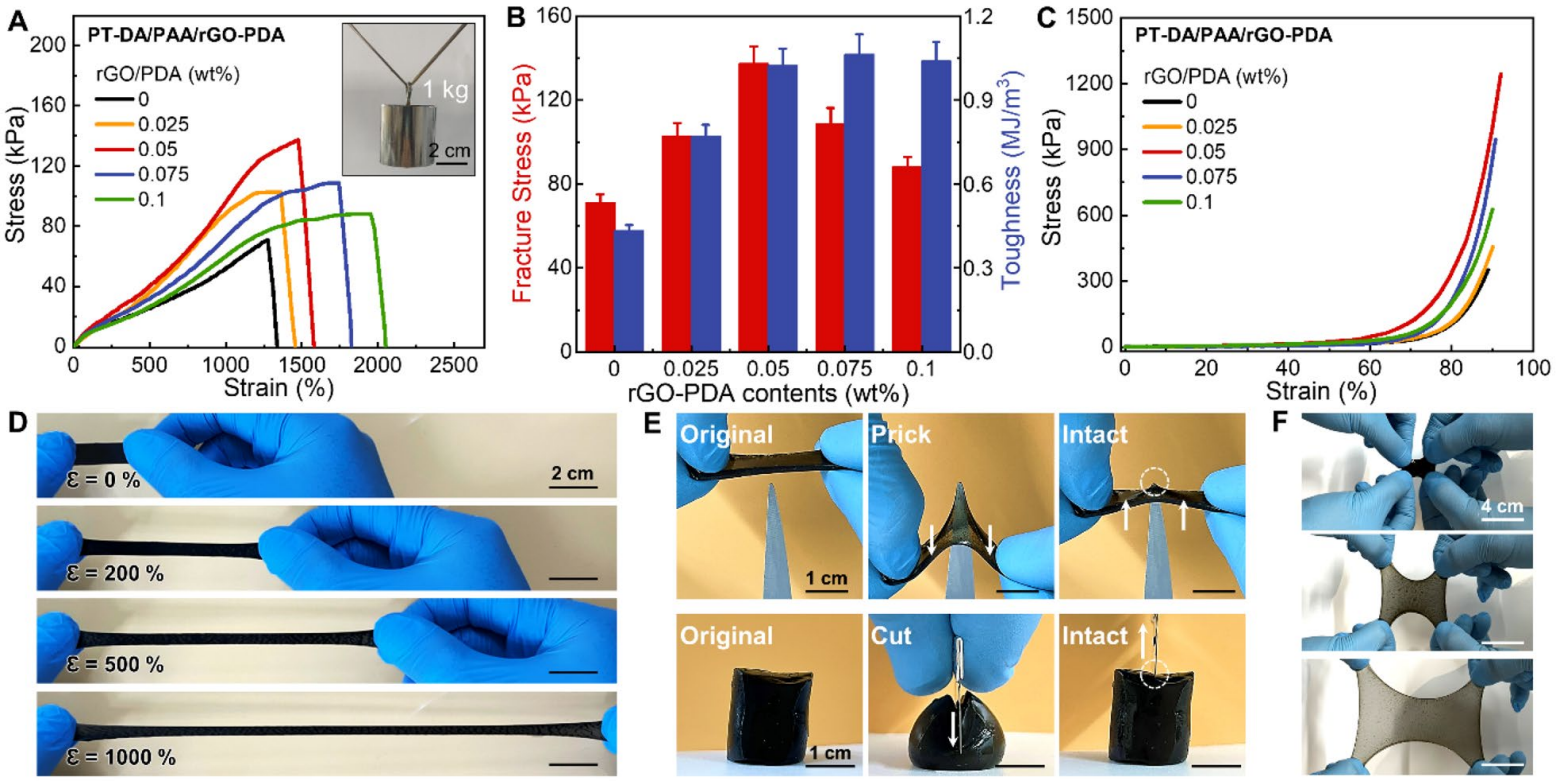
Mechanical characteristics of multifunctional hydrogel. **A** Tensile stress–strain curves of the multifunctional PPGP hydrogel (PT-DA/PAA/rGO-PDA) with varying rGO-PDA concentrations. The inset displays a photograph of the hydrogel successfully supporting a substantial weight of 1 kg without disconnecting. **B** Fracture stress and toughness of multifunctional hydrogels as a function of rGO-PDA content. **C** Compressive stress–strain curves of the multifunctional hydrogel for different rGO-PDA concentrations. **D** Photographs illustrate the remarkable stretchability of the multifunctional hydrogel. **E** Photographs demonstrating the hydrogel’s exceptional mechanical recovery and resilience, even when subjected to highly concentrated stress from sharp objects such as tweezers (top) or a knife (bottom). **F** Photographs displaying the hydrogel’s high stretchability without fracturing, even under biaxial tension with an aerial strain exceeding 600%

Subsequently, we investigated the impact of rGO-PDA loading (0–0.1 wt%) on PT-DA/PAA hydrogels containing 2 wt% PT-DA (Fig. 2A). The inclusion of rGO-PDA content significantly enhanced the mechanical properties of PT-DA/PAA/rGO-PDA (PPGP) hydrogels. Various functional moieties on the surface of rGO-PDA facilitated the formation of hydrogen bonds with PAA and PT-DA chains, thereby enhancing mechanical strength [13, 39, 44]. As the amount of rGO-PDA increased from 0 to 0.05 wt%, both tensile strength and maximum strain of the hydrogels gradually improved. However, increasing the weight percentage of rGO-PDA to 0.1 wt% continued to increase the fracture strain while reducing the fracture strength (Fig. 2A, B). This is because the excessive amount of rGO-PDA hinders the formation of dynamic crosslinking in the hydrogel network due to the strong radical scavenging property of rGO-PDA, consequently diminishing tensile strength but enhancing elongation [45]. These results indicated that at an optimal rGO-PDA content of 0.05 wt%, the multifunctional hydrogels exhibited a substantial elastic elongation of 1590.7 ± 50.0% and an improved fracture strength of 133.3 ± 15.4 kPa. Regarding toughness, the multifunctional PPGP hydrogel demonstrated a significant increase compared to the PT-DA/PAA hydrogel, with no reduction in toughness even at higher rGO-PDA contents (0.075–1.0 wt%) (Fig. 2B). This phenomenon can be attributed to the increasing rGO-PDA contents, which elevated the overall crosslinking density within the hydrogel system and restricted the mobility of polymer segments [46]. We also investigated the compressive strength of the multifunctional hydrogels at various rGO-PDA concentrations (Fig. 2C). Like the tensile test, the hydrogel containing 0.05 wt% rGO-PDA exhibited a maximum compressive stress of 1250 kPa at a strain of 90%, without experiencing any mechanical failure.

Based on these remarkable mechanical properties of the multifunctional hydrogel, we investigated its mechanical deformation and recovery behavior under various conditions (Fig. 2D–G, Additional file 2: Movie S1). During uniaxial stretching, the hydrogel could be elongated by more than 1000% without experiencing any mechanical rupture, and the stretched sample could fully return to its original length after the stress was removed (Fig. 2D). Even when subjected to highly concentrated stress from sharp objects such as tweezers or a knife, the hydrogel demonstrated the ability to regain its pristine state after the stress was removed (Fig. 2E). The hydrogel withstood biaxial tension, achieving an aerial strain of over 600% without fracturing (Fig. 2F). Furthermore, a cylindrical hydrogel with a 3 mm diameter could support a weight of 1 kg without tearing (inset of Fig. 2A). These results illustrate the multifunctional hydrogel’s extraordinary mechanical robustness, enabling its reliable and repeatable utilization in a wide range of wearable and flexible devices and systems (Additional file 2: Movie S1).

### Adhesion behaviors of multifunctional hydrogels

**Fig. 3:**
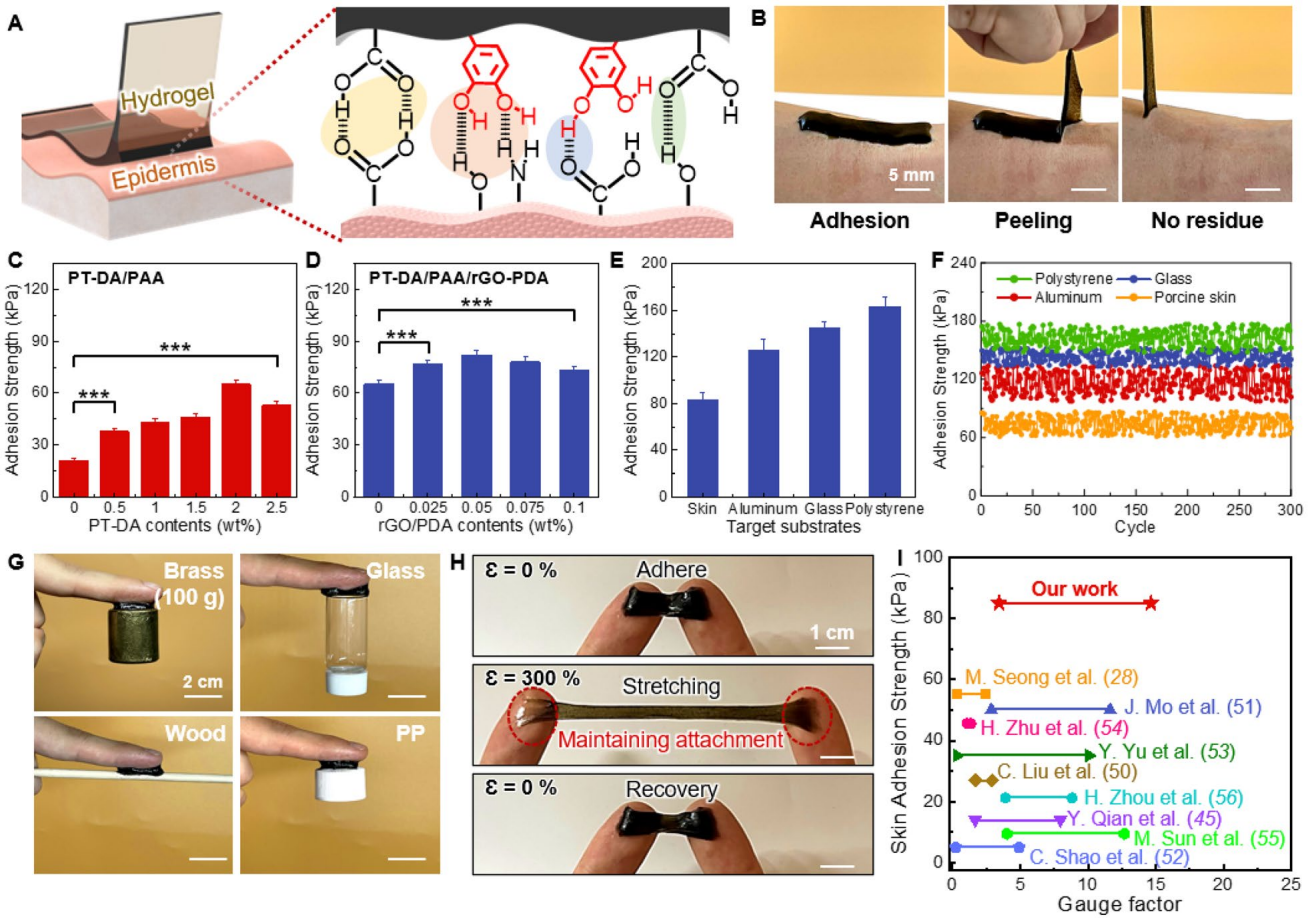
Adhesion properties of multifunctional hydrogels. **A** Schematic illustration depicting the potential adhesion mechanism between the hydrogel and the skin’s epidermis. **B** Photographs demonstrating the robust skin adhesion of the hydrogel. No residues or skin irritation were observed even after 10 cycles of repeated adhering-stripping processes. **C** Adhesion strength of the PT-DA/PAA hydrogels as a function of PT-DA concentrations against porcine skin. **D** Adhesion strength of the multifunctional PPGP (PT-DA/PAA/rGO-PDA) hydrogel as a function of rGO-PDA concentrations (with PT-DA concentration fixed at 2 wt%). **E** Adhesion strengths of the multifunctional hydrogels (PT-DA content: 2 wt%, rGO-PDA content: 0.05 wt%) on various substrates, and **F** their cyclic adhesion performance. **G** Photographs showing the adhesion capability of the multifunctional PPGP hydrogel on a wide range of substrates with varying surface roughness and wettability. **H** Photographs illustrate the PPGP hydrogel’s strong attachment to finger skin without interfacial detachment even during significant stretching (300% strain). **I** A comparison of skin adhesion strength and strain sensitivity (GF) between the PPGP hydrogel and previous hydrogels. The data were analyzed using one-way ANOVA and Tukey’s multiple comparison test (n = 7, ***p < 0.001)

rGO-PDA, PT-DA, and PPA are rich in functional groups such as catechol, hydroxyl, and carboxyl groups. Consequently, the synthesized multifunctional hydrogels can exhibit robust self-adhesion to a wide range of substrates by mimicking a mussel adhesion mechanism (Fig. 3A) [47]. Indeed, the multifunctional hydrogel exhibited strong skin adhesion, leaving no noticeable residue after peel-off (Fig. 3B). This exceptional interfacial adhesion to the skin was attributed to the hydrogen bonds formed between catechol and carboxyl groups on the multifunctional hydrogel and different functional groups (OH, NH_2_, and COOH) on the outermost layers of the skin (Fig. 3A) [48, 49]. To assess the influence of PT-DA on the adhesion properties of the hydrogels, we initially conducted pull-off tests using the PT-DA/PAA hydrogels with varying PT-DA concentrations against porcine skin (Fig. 3C). The pure PAA hydrogel without PT-DA exhibited a skin adhesion strength of 20.7 kPa. Upon adding PT-DA to the PAA hydrogel, skin adhesion increased with rising PT-DA concentrations, reaching a maximum of 65 kPa at 2 wt% PT-DA (Fig. 3C). The improvement in adhesiveness was mainly attributed to the presence of functional catechol and carboxyl groups in the multifunctional hydrogels, which can readily interact with skin surface through hydrogen bonding. However, further increasing the PT-DA content to 2.5 wt% led to a slight reduction in adhesion to 52.8 kPa. This is ascribed to the fact that some aggregations were formed at a high PT-DA concentration.

Next, we evaluated the adhesion strength of the multifunctional PPGP hydrogel as a function of rGO-PDA concentrations (Fig. 3D). We maintained the PT-DA concentration at 2 wt% for this test. Skin adhesion further increased with the incorporation of rGO-PDA into the hydrogel. For example, a slight addition of 0.025 wt% and 0.05 wt% rGO-PDA into the hydrogels resulted in significant adhesion increases to 77 kPa and 85 kPa, respectively (Additional file 3: Movie S2). This is because the molecular interaction between the catechol groups of rGO-PDA and the skin was established [48]. Furthermore, the augmented cohesion induced by adding rGO-PDA also contributed to the improved adhesion capability. However, when the rGO-PDA amount increased to 0.075 wt%, adhesion strength decreased due to rGO-PDA aggregation (Fig. 3D) [48]. During the reduction of GO, part of the catechol groups was oxidized to quinone groups, but the adhesiveness of rGO-PDA was largely retained.

In addition to porcine skin, we also assessed the adhesion strength of the multifunctional PPGP hydrogel on various substrates. As shown in Fig. 3E and Additional file 1: Fig. S6, the hydrogel exhibited substantial adhesion strengths of 126, 145, and 163 kPa for aluminum, glass, and polystyrene substrates, respectively. Moreover, these remarkable adhesion strengths of the hydrogels on diverse substrates were maintained over 300 cycles without noticeable performance degradation (Fig. 3F). Additionally, the multifunctional hydrogel could securely adhere to an array of substrates with varying surface roughness and wettability, including brass, polypropylene, glass, and wood (Fig. 3G). Furthermore, the hydrogel could conformally attach to finger skin without interfacial detachment even during significant stretching (300% strain) (Fig. 3H). Our multifunctional hydrogels demonstrated superior skin adhesion, outstanding stretchability, and remarkable strain sensitivity compared to recently reported mussel-inspired hydrogels (Additional file 1: Table S2, Fig. 3I) [28, 45, 50–56]. The strain sensitivity of the PPGP hydrogel sensor will be discussed in detail in the next section (Sect. 3.4).

### Self-healing and electrical behaviors of multifunctional hydrogels

**Fig. 4:**
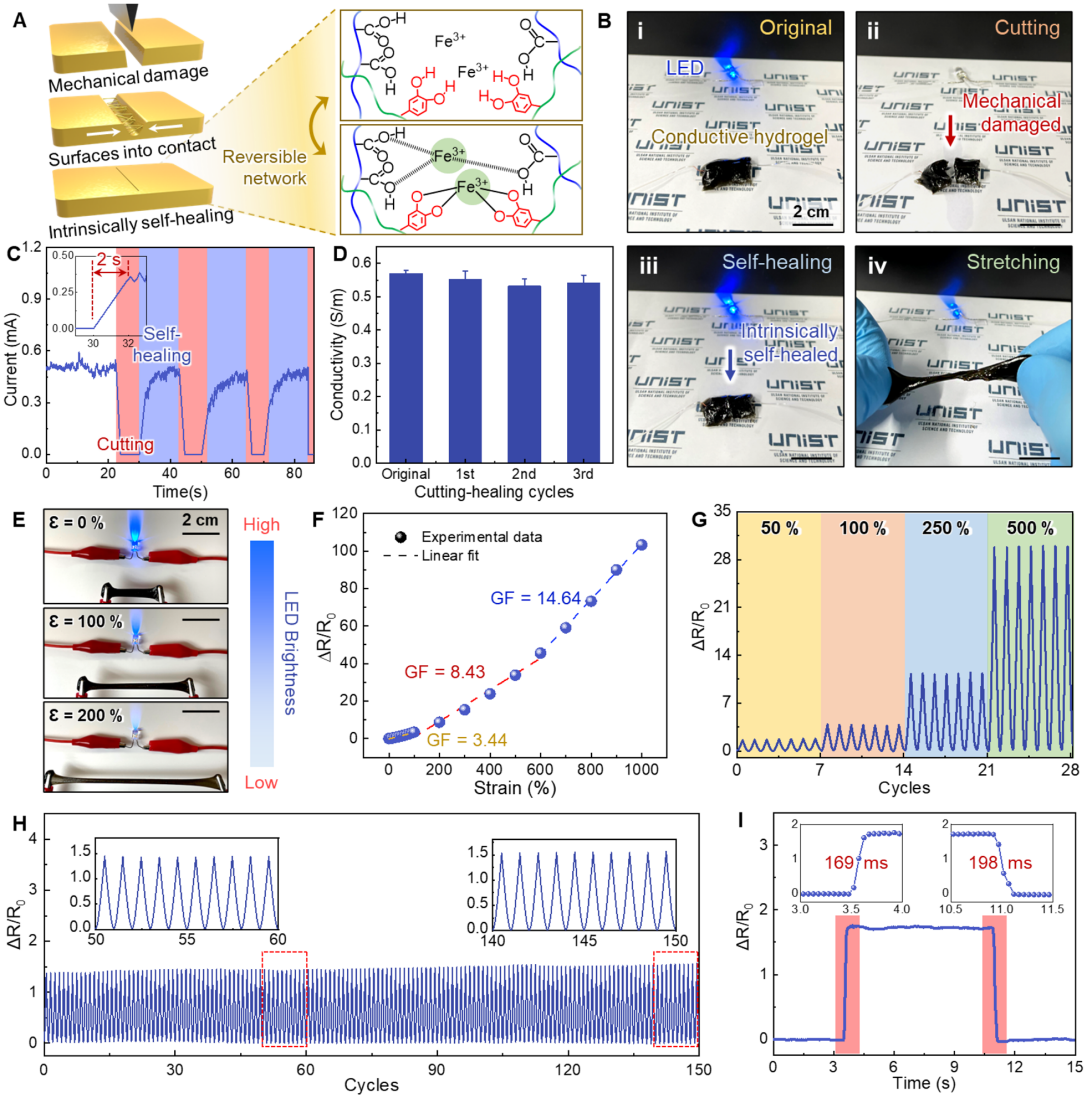
Self-healing and electrical behaviors of multifunctional hydrogels. **A** Schematic illustration depicting the self-healing mechanisms based on the dynamic and reversible crosslinking networks within the hydrogels. **B** Photographs demonstrating the hydrogel’s self-healing property and electrical conductance during a cutting-healing test. **C** Current change of the hydrogel when connected in an electrical circuit during repeating cutting-healing cycles. **D** Electrical conductivity of the hydrogel after undergoing repeating cutting-healing cycles. **E** Photographs illustrating the luminance variations of LED bulbs at different hydrogel elongations (0%, 100%, and 200%). **F** Relative resistance changes of the hydrogel as a function of applied tensile strain. **G** Relative resistance changes of the hydrogels during the application of repeating different strains (ranging from 50 to 500%). **H** Signal stability of the hydrogel during repeated application of 50% strain for 150 cycles. **I** Response time and recovery time of the hydrogel during loading and unloading of tensile strain

Flexible and wearable devices made of soft materials often undergo repeated mechanical deformation, which can result in mechanical or electrical failures [57]. Therefore, self-healing properties can significantly enhance the reliability and long-term durability of wearable devices. In addition to its remarkable stretchability and self-adhesive properties, our multifunctional hydrogel also possesses intrinsic self-healing capabilities. Figure 4A illustrates the schematic of the self-healing process and the corresponding molecular structures of the multifunctional PPGP hydrogels. These hydrogels exhibit numerous dynamic interactions within their networks, and these dynamic bonds confer them with spontaneous healing abilities. For instance, dynamic metal coordination interactions involving Fe^3+^, catechol, and carboxylic groups facilitate the free movement of ferric ions, allowing for the rapid recombination of the hydrogels solely through physical contact (Fig. 4A) [31].

To assess the hydrogel’s detailed self-healing capability and electrical properties, we integrated the multifunctional hydrogel into a simple circuit with an LED indicator (Fig. 4B). Initially, the hydrogel efficiently conducted current to the LED, keeping it illuminated (Fig. 4B-i). The LED immediately turned off when the hydrogel was cut into two parts (Fig. 4B-ii). However, upon reconnection of the separated hydrogel sections, they rapidly self-healed, restoring the current flow and re-illuminating the LED even when subjected to extensive stretching (Fig. 4B-iii, iv). It’s worth noting that simple physical contact allows for swift mechanical and electrical reconnections and self-healing, eliminating the need for additional heat or solvent applications. We measured the current passing through the circuit for a quantitative assessment of self-healing and electrical performance. Initially, the current was 0.5 mA. Cutting the hydrogel resulted in zero current through the circuit. However, upon reuniting the divided hydrogel sections through physical contact, the current rapidly increased to 72% of its original level within 2 s and reached 94% within 5 s. This exceptional electrical self-healing performance remained consistent through multiple cutting-healing cycles (Fig. 4C). The hydrogel also maintained its original electrical conductivity of 0.562 S m^−1^ with minimal material degradation even after several cutting-healing processes (Fig. 4D) [46]. We also investigated the mechanical self-healing properties of the PPGP hydrogel by evaluating its mechanical recovery after self-healing. To assess this, we divided the hydrogel specimens into two equal sections and brought them into contact for varying durations before performing tensile tests. The results demonstrated that PPGP hydrogel regains its mechanical properties more effectively with longer contact times. For instance, the hydrogel recovered 42.5% of its original strength after 30 min of contact. When the contact duration increased to 120 min, the PPGP hydrogel recovered 91.6% of its original mechanical strength. (Additional file 1: Fig. S7).

As depicted in Fig. 4E, the LED bulb’s brightness dimmed as the hydrogel strain gradually increased from 0 to 200% (Additional file 4: Movie S3). Remarkably, the original brightness was instantly restored upon releasing the applied stress, demonstrating the hydrogel’s excellent strain response. Consequently, we calculated the GF of the hydrogel for various applied strains (Fig. 4F). GF is a key performance metric for quantitatively assessing the sensitivity of a strain sensor, defined as (ΔR/R_0_)/ε, where ΔR and R represent the change in resistance caused by strain and the initial resistance, respectively [58–61]. Due to the remarkable stretchability of the hydrogel, the hydrogel strain sensor could be effectively utilized for a wide range of strain detection (0–1000%). Within this strain range, the PPGP hydrogel exhibited three distinct regions with different slopes in its stress–strain curve. This behavior is attributed to the nonlinear deformation behavior of the hydrogel networks under varying levels of strain [62, 63]. In the low strain range (0–150%), the PPGP hydrogel exhibited a GF of 3.4 and a linearity (*R*^2^) of 0.9961. For the intermediate strain range (150–650%), the GF increased to 8.43 with a corresponding *R*^2^ of 0.9924. In the high strain range (650–1000%), the GF and *R*^2^ were 14.64 and 0.9987, respectively, indicating the high sensitivity of the PPGP strain sensors compared to existing hydrogel sensors (Additional file 1: Table S2, Fig. 3I) [28, 45, 50–54]. This high GF is attributed to the microporous structure of the PPGP hydrogel [46, 64]. Furthermore, when subjected to dynamic strains within various ranges (0–500%), the hydrogel sensor demonstrated consistent strain sensitivity and repeatability (Fig. 4G). Sensing repeatability and stability were also observed during repeated stretching tests over 150 cycles (Fig. 4H). Additionally, the sensor exhibited a fast response time (169 ms) and recovery time (198 ms), due to the outstanding elastic and resilient properties of the hydrogel (Fig. 4I).

### Multifunctional hydrogel sensors for physiological and motion monitoring

**Fig. 5:**
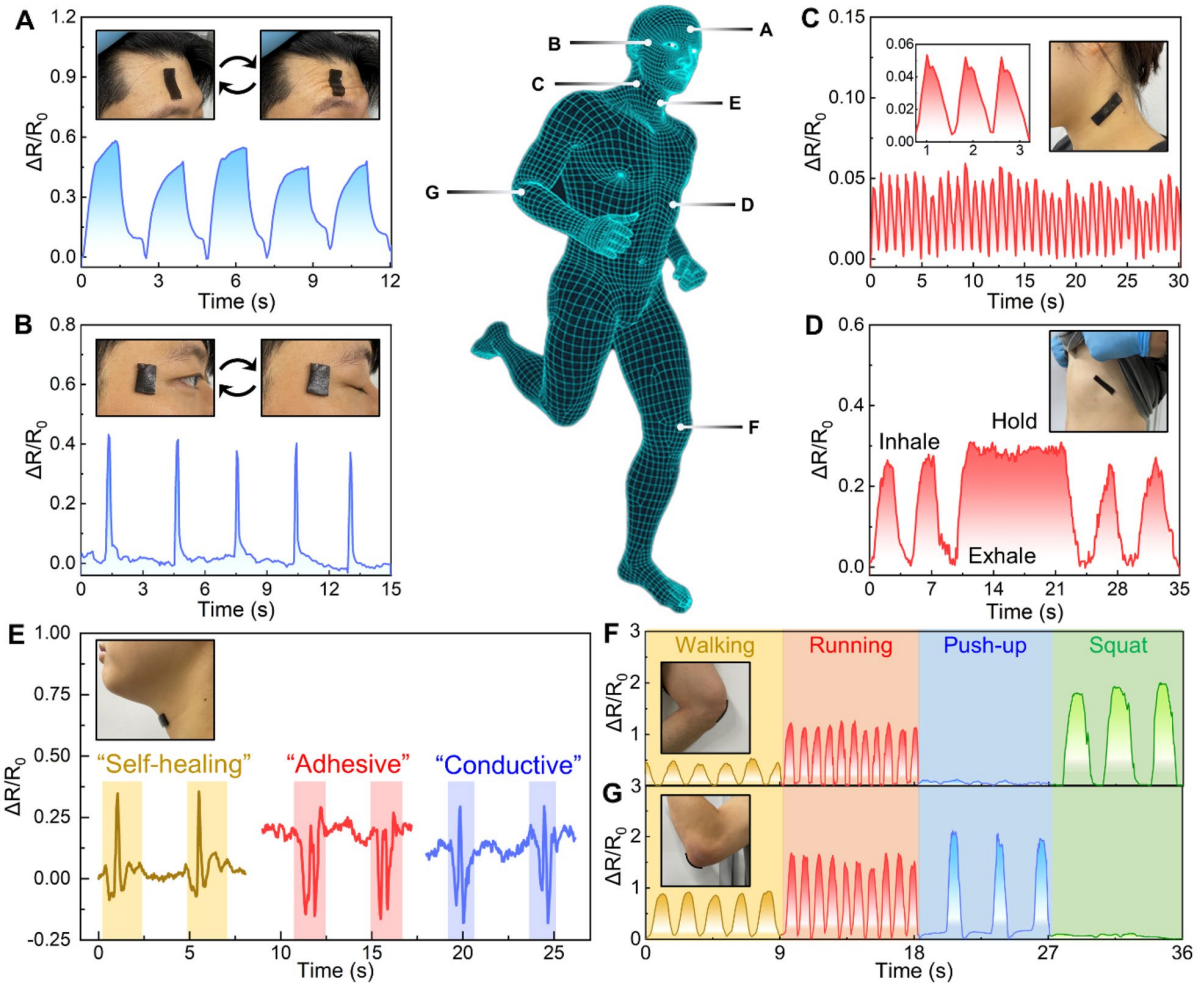
Multifunctional hydrogel-based wearable strain sensors. **A**–**D** Relative resistance changes are measured by the hydrogel sensor fixed onto various regions of the human body including: **A** forehead, **B** outer canthus, **C** side neck, and **D** chest. These sensors could accurately detect **A** forehead movements, **B** eye blinking, **C** carotid pulsations, and **D** respiratory activity. **E** Measured resistance changes of the sensor when the volunteer speaks “self-healing,” “adhesive,” and “conductive.” **F** Real-time resistance changes of the sensor attached to the knee joint during activities such as walking, running, push-ups, and squatting. **G** Real-time resistance changes of the sensor attached to the elbow joint during walking, running, push-ups and squatting

The multifunctional hydrogel, owing to its exceptional qualities of robust interfacial adhesion, remarkable stretchability, self-healing capabilities, and high sensitivity, presents a promising platform for wearable sensors to monitor diverse human activities. To illustrate the practical utility of these hydrogel sensors, we initially affixed them to two specific anatomical locations: the forehead (Fig. 5A) and the outer canthus (corner of the eye) (Fig. 5B). These placements enabled precise and repetitive detection of eyebrow elevation, forehead muscle movements, and eye blinking, with the respective relative resistance change values aligning closely with the specific motions observed.

Upon attaching the hydrogel sensor to the neck lateral, we accurately measured physiological pulse (Fig. 5C). The recorded pulse rate, approximately 66 times per minute, closely mirrored readings obtained from a commercially available pulse meter. Furthermore, our sensor exhibited tendencies associated with diastolic and systolic blood pressure [65], demonstrating its remarkable sensing and detection capabilities. Additionally, when applied to the chest region, our sensors successfully detected inhalation and exhalation patterns in the human body. Figure 5E shows the hydrogel sensor’s placement on the throat, where it demonstrated the ability to capture electrical signals generated during the articulation of distinct words such as “self-healing,” “adhesive,” and “conductive” [66]. The observable variations in signal patterns between different words accentuate the sensor’s remarkable potential for enhancing human–machine interactions. Beyond capturing subtle bodily movements, our strain sensors also demonstrate exceptional competence in monitoring larger limb motions, particularly those involving the knees and elbows, rendering them well-suited for athletic applications. When affixed to these joints, the sensors provided real-time tracking of dynamic body movements, sustaining reliable adhesion without detachment, courtesy of the substantial stretchability and robust skin adhesion capabilities inherent to the multifunctional hydrogel (Fig. 5F).

## Conclusion

In summary, we have presented a multifunctional hydrogel with remarkable properties, including high stretchability, self-adhesion, self-healing capabilities, and strain sensitivity. This versatile hydrogel was synthesized through a simple free radical polymerization of PT-DA, AA, and rGO-PDA. The resulting hydrogel incorporates various dynamic interactions, such as hydrogen and coordination bonds, within its network structure, endowing it with remarkable characteristics. These include impressive stretchability, capable of reaching up to 2000%, robust self-adhesive properties on a variety of substrates (including skin, brass, rubber, polypropylene, iron, glass, and wood), and rapid self-healing abilities at room temperature within a few seconds. Notably, the hydrogel exhibited an impressive skin adhesion strength of 85 kPa, surpassing many existing hydrogel adhesives. Moreover, it displayed highly sensitive responsiveness to strain. Leveraging its outstanding strain sensitivity, strong skin adhesion, and high stretchability, we harnessed this multifunctional hydrogel as an advanced wearable sensor. This sensor demonstrated its ability to adhere to various regions of the human body securely, enabling the detection of subtle physiological signals and real-time body movements with noticeable sensitivity (maximum GF: 14.6), an extensive sensing range (up to 1000%), high repeatability (> 150 cycles), and rapid response times (169 ms).

Several previous studies have also reported on stretchable, self-adhesive, and conductive hydrogels. However, combining several exceptional functionalities, such as high stretchability, strong interfacial adhesion, outstanding self-healing capability, and sensitivity, into a single material poses significant technical challenges. For instance, Gao et al. developed an ultra-adhesive hydrogel [67]. The hydrogel exhibited high stretchability (> 2000%), good electrical conductivity (up to 1.95 S m^–1^), and high skin adhesion (957 N m^–1^). However, they showed low self-healing efficiency (52%) even after 24 h of healing. Chen et al. also reported an ultra-stretchable and sticky hydrogel [68]. The hydrogel showed high stretchability (> 1900%), good electrical conductivity (0.39 S m^–1^), and high skin adhesion (2126 J m^–2^). However, their self-healing properties have not been investigated, and the adhesion repeatability of these hydrogels has not been demonstrated. In contrast, the PPGP hydrogel proposed in this study exhibited a remarkable combination of outstanding functionalities, including rapid self-healing, strong and repeatable self-adhesion, exceptional stretchability, and high strain sensitivity. Notably, the PPGP hydrogel showed rapid self-healing capacity (94% electrical recovery in 5 s and 91.6% mechanical recovery in 2 h) without requiring any external energy input. This is significantly faster than that reported in the work by Gao et al. (52% mechanical recovery in 24 h). Furthermore, the PPGP hydrogel could maintain a high level of skin adhesion during repeated cycles of attachment and detachment over 300 without any degradation (Fig. 3F).

However, it is important to note that most hydrogels tend to dehydrate under ambient conditions, ultimately affecting their adhesion and sensing performance in long-term applications [69]. The PPGP hydrogel also exhibited an 18.6% decrease in skin adhesion strength to 69.2 kPa, while the sensitivity decreased to 2.84 under 100% strain after exposure to ambient conditions (25 °C) for 24 h. Therefore, it is necessary to address the inherent issue of hydrogel drying for the long-term stability of hydrogel sensors. To tackle this challenge, recent studies have explored various techniques, including the incorporation of inorganic particle additives, salt treatment, and the use of polyols solvent to enhance water retention capacity [70]. Additionally, protective coatings applied to the hydrogel surface can help retard water evaporation. By integrating these strategies, we believe our multifunctional hydrogel holds immense potential to significantly contribute to the development of advanced hydrogel-based wearable devices and systems across a broad spectrum of applications, including healthcare, soft robotics, and human–machine interactions.

### Supplementary Information

**Additional file 1: Figure S1.** (A) Synthesis of pectin-dopamine conjugation (PT-DA), (B) ^1^H NMR spectra of pectin, dopamine, and PT-DA conjugate, and (C) FT-IR spectra of pectin, dopamine, and PT-DA conjugate. **Figure S2.** XRD patterns of (A) GO and (B) rGO-PDA. **Figure S3.** XPS Spectra recorded for the C 1s fitting in (A) GO and (B) rGO-PDA. **Figure S4.** Cross-sectional SEM images of (A) PAA, (B) PT-DA/PAA, and (C) PT-DA/PAA/rGO-PDA hydrogels. **Figure S5.** (A) Photograph showing the PT-DA/PAA hydrogels exhibiting extraordinary mechanical properties. (B) Tensile stress–strain curves of PT-DA/PAA hydrogels as a function of PT-DA. **Figure S6.** Adhesion curve of the PPGP hydrogel against different substrates. **Figure S7.** Mechanical self-healing properties of the PPGP hydrogels. (A) Photographs demonstrating the mechanical self-healing ability of the PPGP hydrogels for different contact durations (5 and 60 min). (B) Stress–strain curves of the original and recovered hydrogel samples with different contact times (30, 60, and 120 min). (C) Mechanical self-healing efficiency of the PPGP hydrogel as a function of contact time. The self-healing efficiency is defined as the ratio of fracture strength between recovered and original hydrogels.

**Additional file 2:** **Movie S1.** Mechanical recovery and resilience properties of the PPGP hydrogel.

**Additional file 3:** **Movie S2.** Skin adhesion performance of the PPGP hydrogel.

**Additional file 4:** **Movie S3.**Strain response characteristics of the PPGP hydrogel sensor.

## Data Availability

The data that support the findings of this study are available from the corresponding author on reasonable request.
