# Supplementary material for: Mussel-inspired resilient hydrogels with strong skin adhesion and high-sensitivity for wearable device
Source: Nano Converg. 2024 Mar 21;11:12. doi: 10.1186/s40580-024-00419-4 (PMC10957857; doi:10.1186/s40580-024-00419-4)
Supplement: Supplementary file 1 — Additional file 1: Figure S1. (A) Synthesis of pectin-dopamine conjugation (PT-DA), (B) 1H NMR spectra of pectin, dopamine, and PT-DA conjugate, and (C) FT-IR spectra of pectin, dopamine, and PT-DA conjugate. Figure S2. XRD patterns of (A) GO and (B) rGO-PDA. Figure S3. XPS Spectra recorded for the C 1s fitting in (A) GO and (B) rGO-PDA. Figure S4. Cross-sectional SEM images of (A) PAA, (B) PT-DA/PAA, and (C) PT-DA/PAA/rGO-PDA hydrogels. Figure S5. (A) Photograph showing the PT-DA/PAA hydrogels exhibiting extraordinary mechanical properties. (B) Tensile stress–strain curves of PT-DA/PAA hydrogels as a function of PT-DA. Figure S6. Adhesion curve of the PPGP hydrogel against different substrates. Figure S7. Mechanical self-healing properties of the PPGP hydrogels. (A) Photographs demonstrating the mechanical self-healing ability of the PPGP hydrogels for different contact durations (5 and 60 min). (B) Stress–strain curves of the original and recovered hydrogel samples with different contact times (30, 60, and 120 min). (C) Mechanical self-healing efficiency of the PPGP hydrogel as a function of contact time. The self-healing efficiency is defined as the ratio of fracture strength between recovered and original hydrogels. [file 40580_2024_419_MOESM1_ESM.docx]

**Additional file 1**

Mussel-inspired resilient hydrogels with strong skin adhesion and high-sensitivity for wearable device

Stalin Kondaveeti^1,3‡^, Geonjun Choi^1‡^, Sarath Chandra Veerla^1^, Somi Kim^1^, Jaeil Kim^1^, Hee Jin Lee^2^, Unnikrishnan Kuzhiumparambil^3^, Peter J Ralph^3^, Junyeob Yeo^2^*, and Hoon Eui Jeong^1^*

^1^Department of Mechanical Engineering, Ulsan National Institute of Science and Technology (UNIST), Ulsan, 44919, Republic of Korea

^2^Department of Physics, Kyungpook National University, 80 Daehak-ro, Bukgu, Daegu, 41566, Republic of Korea

^3^Climate Change Cluster, University of Technology Sydney, Ultimo, NSW, Australia

*Corresponding Authors: Hoon Eui Jeong ([hoonejeong@unist.ac.kr](mailto:hoonejeong@unist.ac.kr)), Junyeob Yeo ([junyeob@knu.ac.kr](mailto:junyeob@knu.ac.kr))

^‡^These authors contributed equally to this work.

**Table S1.** Compositions of multifunctional PAA, PT-DA/PAA and PT-DA/PAA/rGO-PDA hydrogels.

| **Hydrogels** | **PT-DA**  **(g)** | **AA (g)** | **rGO-PDA**  **(g)** | **FeCl_3_.6H_2_O** **(g)** | **APS**  **(g)** | **H_2_O**  **(mL)** |
| --- | --- | --- | --- | --- | --- | --- |
| PAA | - | 10 | - | 0.025 | 0.05 | 90 |
| PT-DA 0.5/PAA | 0.5 | 10 | - | 0.025 | 0.05 | 90 |
| PT-DA 1/PAA | 1 | 10 | - | 0.025 | 0.05 | 90 |
| PT-DA 1.5/PAA | 1.5 | 10 | - | 0.025 | 0.05 | 90 |
| PT-DA 2/PAA | 2 | 10 | - | 0.025 | 0.05 | 90 |
| PT-DA 2.5/PAA | 2.5 | 10 | - | 0.025 | 0.05 | 90 |
| PT-DA 2/PAA/rGO-PDA 0.025 | 2 | 10 | 0.025 | 0.025 | 0.05 | 90 |
| PT-DA 2/PAA/rGO-PDA 0.05 | 2 | 10 | 0.05 | 0.025 | 0.05 | 90 |
| PT-DA 2/PAA/rGO-PDA 0.075 | 2 | 10 | 0.075 | 0.025 | 0.05 | 90 |
| PT-DA 2/PAA/rGO-PDA 0.1 | 2 | 10 | 0.1 | 0.025 | 0.05 | 90 |

**Table S2.** Comparison of multifunctional performance between the proposed mussel-inspired hydrogel and the existing hydrogels.

| **Hydrogel network** | **Maximum strain (%)** | **Skin adhesion**  **(kPa)** | **Conductivity**  **(S m^-1^)** | **Gauge factor** | **Self-healing time**  **(s)** | **Refs** |
| --- | --- | --- | --- | --- | --- | --- |
| PT-DA/PAA/rGO-PDA conductive hydrogel | 2000% | 85 ^a^ | 0.56 | 3.44 (0–150%) 8.43 (150-650%) 14.64 (650–1000%) | 2^c^ | This work |
| Poly(acrylic acid)/  Cellulose nanocrystals/ Tannic acid | 2952% | 5^a^ | – | 0.23 (0–40%) 4.90 (65–75%) | 600^d^  (10 min) | [1] |
| PAA-rGO/PDA | 600% | – | – | 0.31(100%) 1.32(500%) | 14400^d^  (4 h) | [2] |
| Acrylamide/  N-(3-aminopropyl) methyl acrylamide hydrochloride/PDA/rGO | 1156.9% | 8.2^b^ | 0.12 | 5.4 (100%) | 1.21^c^ | [3] |
| Poly(acrylic acid)/ Tannic acid/ Hemicellulose | 5600% | 8.5^a^ | 0.025 | – | 7200^d^  (2 h) | [4] |
| P(MArg-FHVI-AA) | 2613% | 37^b^ | 0.39 | 1.6 (100%)  3.7 (200%) | 3600^d^  (1 h) | [5] |
| CPVA/GO-PDA | 1457 % | 35.06^b^ | 0.34 | 0.33(0–75%)  0.90(75–150%) 5.52 (150–350%)  10.07(350-500%) | – | [6] |
| Polyvinyl alcohol (PVA)/ Poly(dopamine)/ Carbon nanotube(CNT) | – | 45.6^a^ | – | 1.08(0–100%)  1.04(100–200%) 1.35 (200–300%) | 5^c^ | [7] |
| Nano-polydopamine/  Reinforced hemicellulose | 100% | 4.1^b^ | 0.36 | 1.04(0–45%) | – | [8] |
| Poly(vinyl alcohol)/ Pectin/Tannic acid | – | 55^a^ | 4.7 | 0.4 (0–600 %) 2.5 (1400–2000 %) | 3^c^ | [9] |
| PDA/sodium alginate/  Polyacrylamide | – | 24.5^a^ | – | – | – | [10] |
| GO-PVA-PAA-N-hydroxysuccinimide ester (NHS ester) | 200% | 60^b^ | 2.6 | – | – | [11] |
| Polyacrylamide/sodium alginate/EGaIn-CNT | 2200% | 9.45^b^ | 94 | 4.8 (0–200%)  12.7 (200–600%) | 1200 (20 min) | [12] |
| poly(acrylic acid-co-acrylamide)/amylopectin | 1089% | 21.16^b^ | 0.49 | 3.91 (250%)  8.82 (1000%) | - | [13] |
| PAA- NHS ester/gelatin | 1600% | 120 ^a^ | – | – | – | [14] |

^a^Maximum adhesion strength in normal adhesion test

^b^Maximum adhesion strength in lap-shear test

^c^Recovery time for electrical properties during self-healing process

^d^Recovery time for mechanical properties (> 90 % of recovery ratio) during self-healing process

**
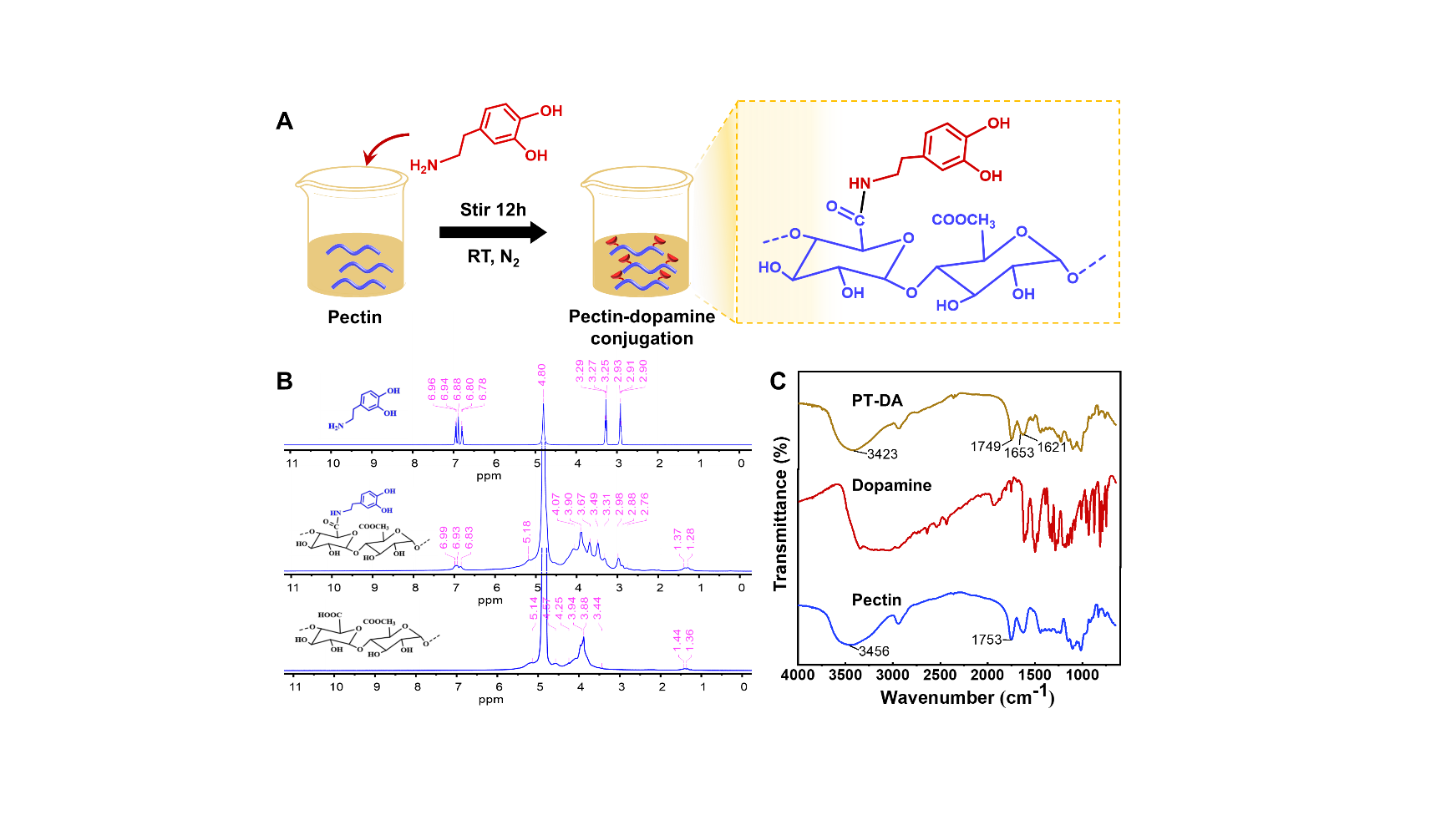
**

**Fig. S1.** (A) synthesis of pectin-dopamine conjugation (PT-DA), (B) ^1^H NMR spectra of pectin, dopamine, and PT-DA conjugate, and (C) FT-IR spectra of pectin, dopamine, and PT-DA conjugate.


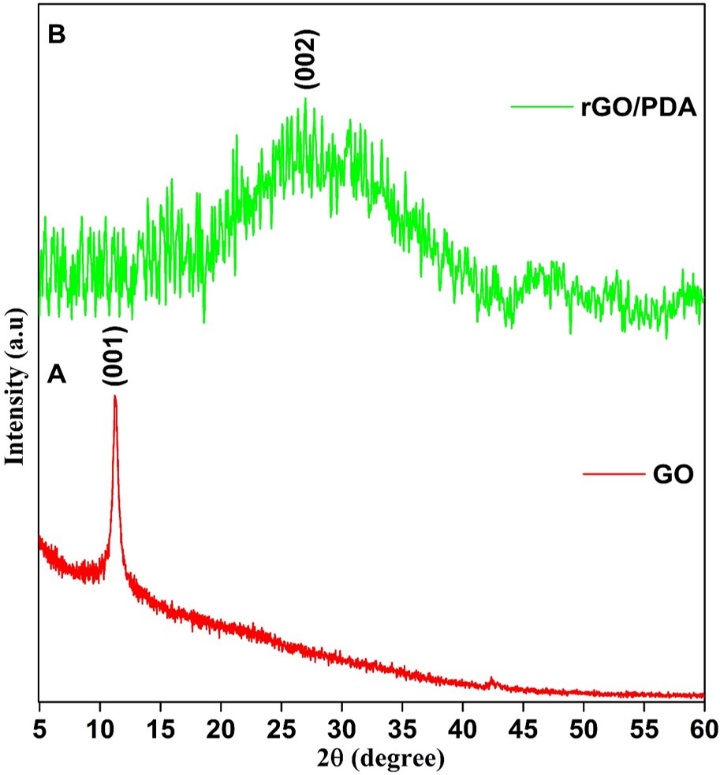


**Fig. S2.** XRD patterns of (A) GO and (B) rGO-PDA


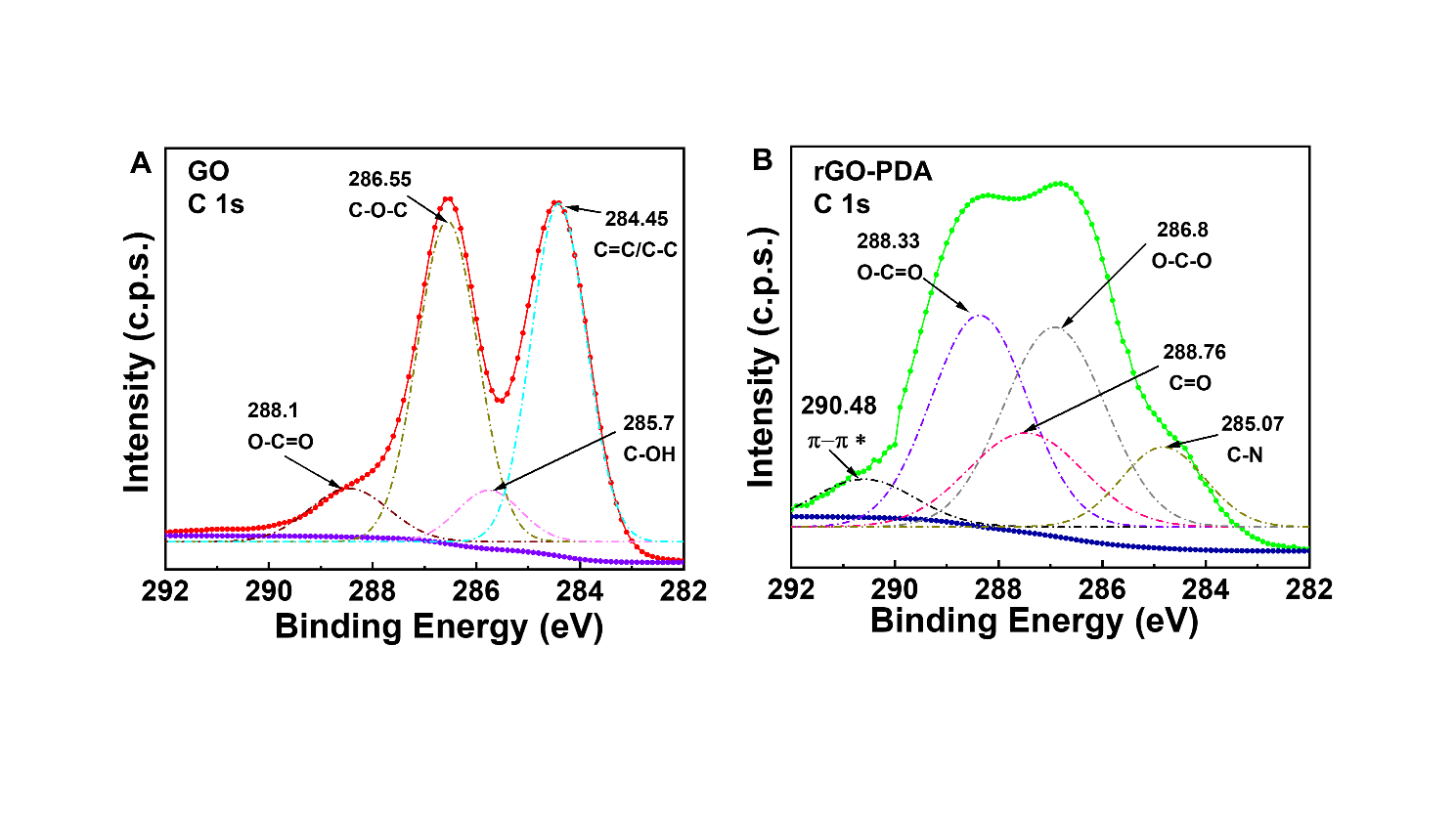


**Fig. S3.** XPS Spectra recorded for the C 1s fitting in (A) GO and (B) rGO-PDA


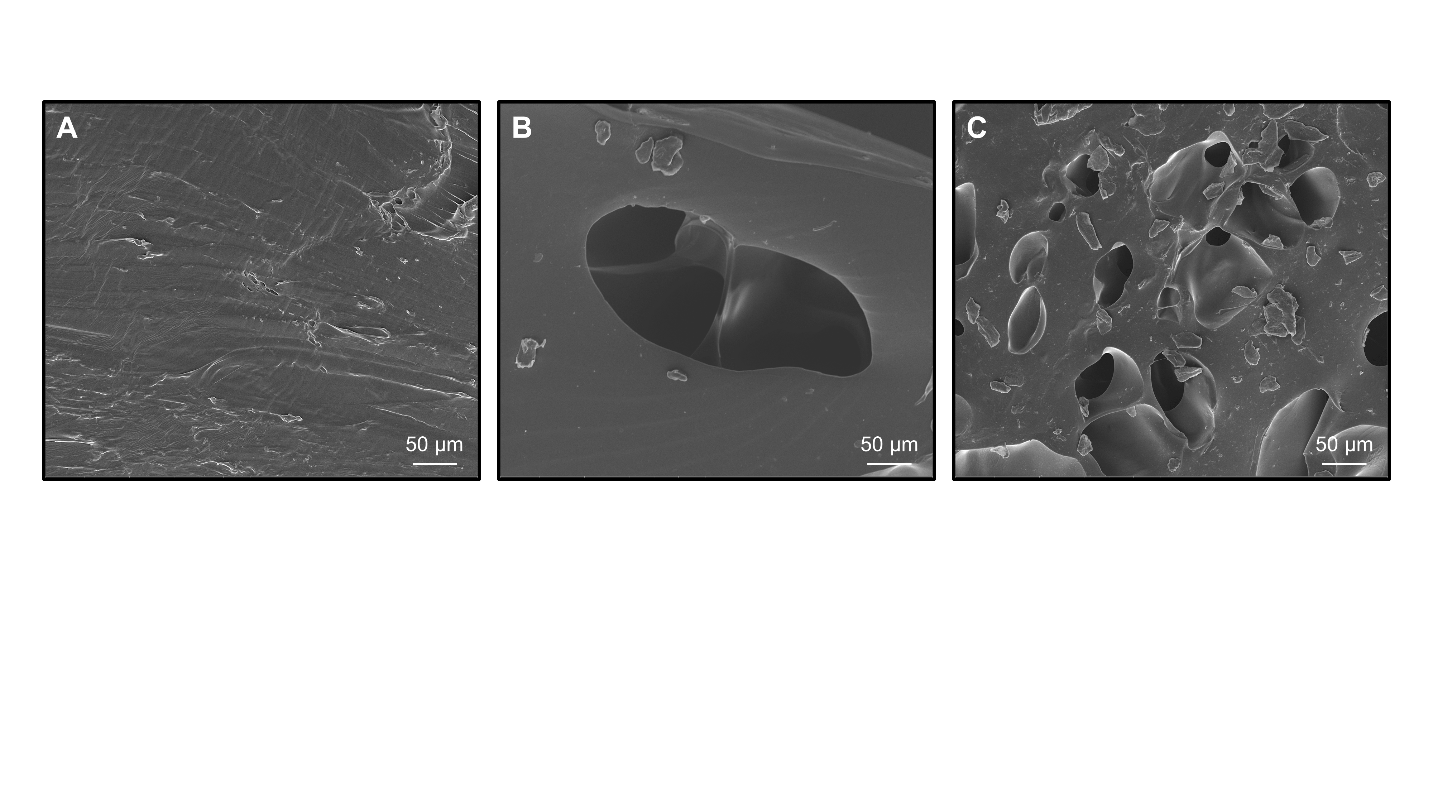


**Fig. S4.** Cross-sectional SEM images of (A) PAA, (B) PT-DA/PAA, and (C) PT-DA/PAA/rGO-PDA hydrogels.


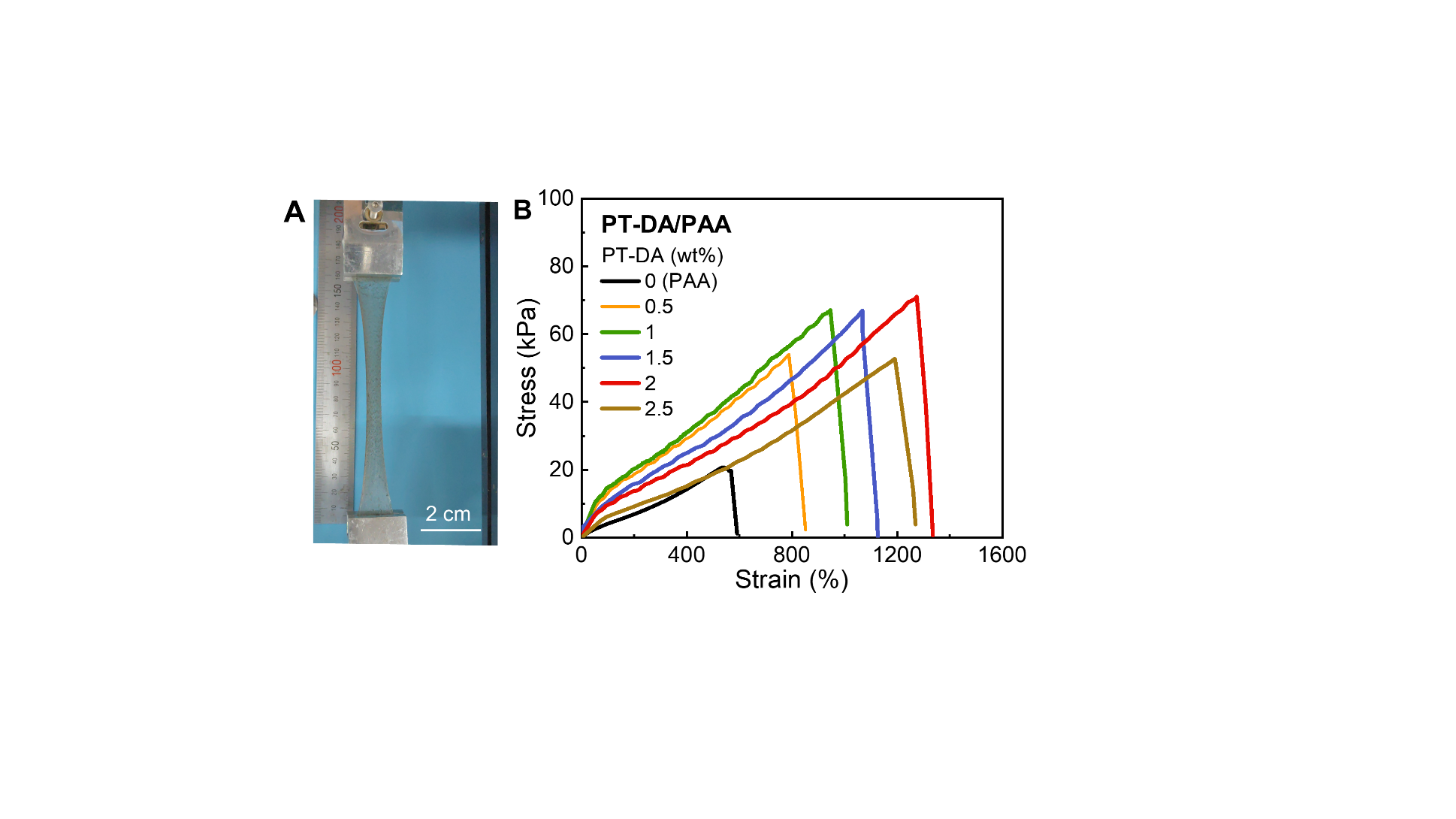


**Fig. S5.** (A) Photograph showing the PT-DA/PAA hydrogels exhibiting extraordinary mechanical properties. (B) Tensile stress–strain curves of PT-DA/PAA hydrogels as a function of PT-DA.


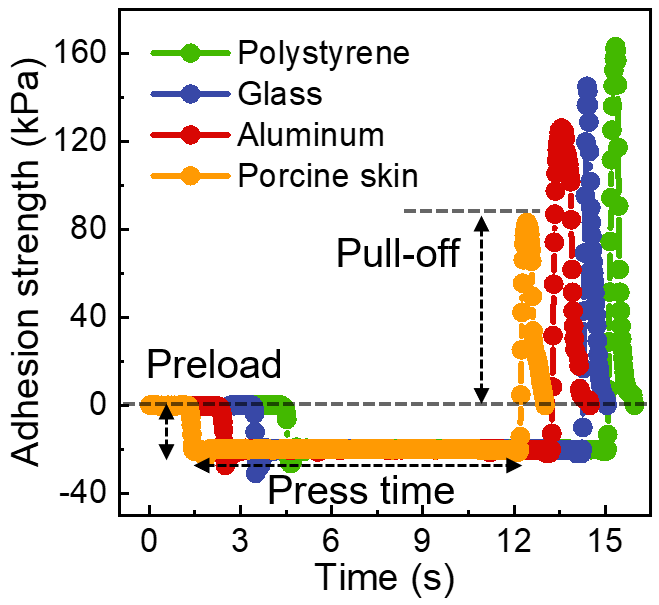


**Fig. S6.** Adhesion curve of the PPGP hydrogel against different substrates.


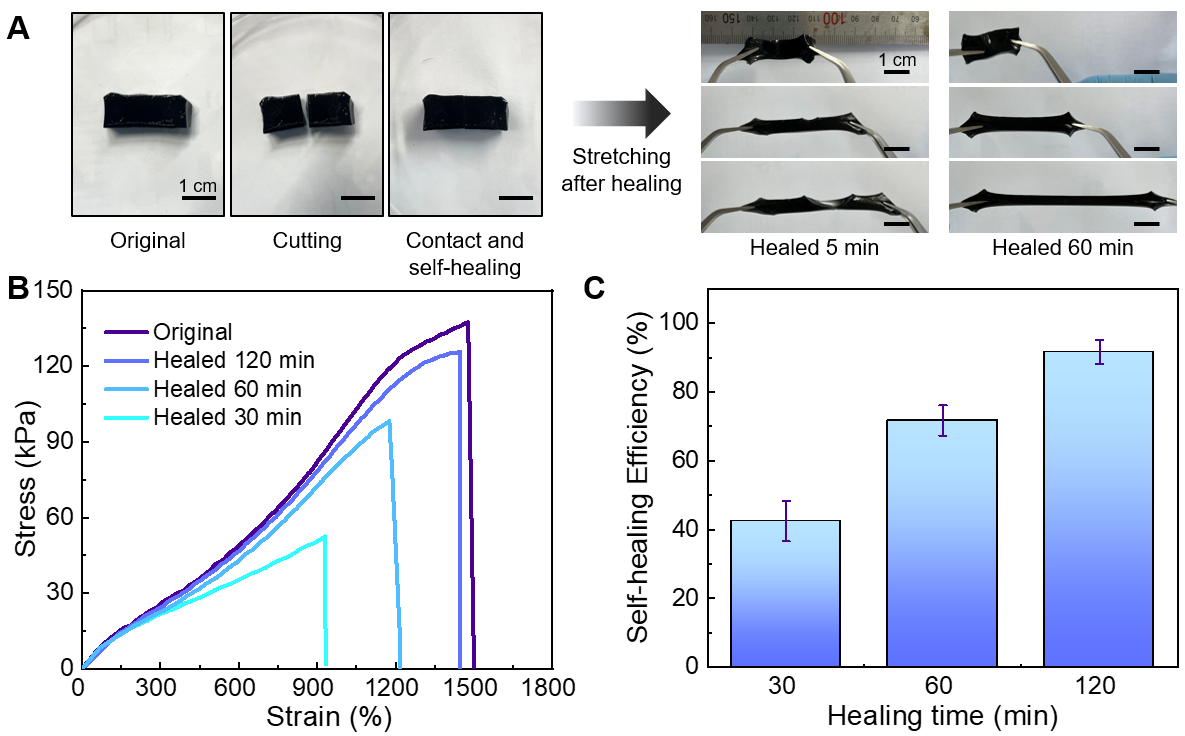


**Fig. S7.** Mechanical self-healing properties of the PPGP hydrogels. (A) Photographs demonstrating the mechanical self-healing ability of the PPGP hydrogels for different contact durations (5 and 60 minutes). (B) Stress–strain curves of the original and recovered hydrogel samples with different contact times (30, 60, and 120 minutes). (C) Mechanical self-healing efficiency of the PPGP hydrogel as a function of contact time. The self-healing efficiency is defined as the ratio of fracture strength between recovered and original hydrogels.

**References**

[1] C. Shao, M. Wang, L. Meng, H. Chang, B. Wang, F. Xu, J. Yang, P. Wan, Mussel-Inspired Cellulose Nanocomposite Tough Hydrogels with Synergistic Self-Healing, Adhesive, and Strain-Sensitive Properties, Chem. Mater. 30 (2018) 3110-3121. <https://doi.org/10.1021/acs.chemmater.8b01172>.

[2] X. Jing, H.-Y. Mi, X.-F. Peng, L.-S. Turng, Biocompatible, self-healing, highly stretchable polyacrylic acid/reduced graphene oxide nanocomposite hydrogel sensors via mussel-inspired chemistry, Carbon 136 (2018) 63-72. <https://doi.org/10.1016/j.carbon.2018.04.065>.

[3] R. Zhao, X.Y. Xu, L. Hu, Highly Strong, Stretchable, and Conductive Reduced Graphene Oxide Composite Hydrogel-Based Sensors for Motoring Strain and Pressure, ACS Appl. Polym. Mater. 3 (2021) 5155-5161. <https://doi.org/10.1021/acsapm.1c00898>.

[4] X.Q. Gong, C.L. Fu, N. Alam, Y.H. Ni, L.H. Chen, L.L. Huang, H.C. Hu, Preparation of Hemicellulose Nanoparticle-Containing Ionic Hydrogels with High Strength, Self-Healing, and UV Resistance and Their Applications as Strain Sensors and Asymmetric Pressure Sensors, Biomacromolecules 23 (2022) 2272-2279. <https://doi.org/10.1021/acs.biomac.1c01640>.

[5] H. Zhang, J.D. He, T. Peng, J.Q. Qu, Ultra-stretchable, Antifatigue, Adhesive, and Self-Healing Hydrogels Based on the Amino Acid Derivative and Ionic Liquid for Flexible Strain Sensors, ACS Appl. Polym. Mater. 4 (2022) 7575-7586. <https://doi.org/10.1021/acsapm.2c01241>.

[6] Y. Yu, X. Zhao, L. Ye, A novel biocompatible wearable sensors based on poly (vinyl alcohol)/graphene oxide hydrogel with superior self-adhesion, flexibility and sensitivity, Compos. Struct. 309 (2023) 116768. <https://doi.org/10.1016/j.compstruct.2023.116768>.

[7] H. Zhu, J. Xu, X. Sun, Q. Guo, Q. Guo, M. Jiang, K. Wu, R. Cai, K. Qian, Wearable, fast-healing, and self-adhesive multifunctional photoactive hydrogel for strain and temperature sensing, J. Mater. Chem. A 10 (2022) 23366-23374. <https://doi.org/10.1039/d2ta06072h>.

[8] Y. Li, M.Z. Yao, Y.D. Luo, J. Li, Z.L. Wang, C. Liang, C.R. Qin, C.X. Huang, S.Q. Yao, Polydopamine-Reinforced Hemicellulose-Based Multifunctional Flexible Hydrogels for Human Movement Sensing and Self-Powered Transdermal Drug Delivery, ACS Appl. Mater. Interfaces 15 (2023) 5883–5896. <https://doi.org/10.1021/acsami.2c19949>.

[9] M. Seong, S. Kondaveeti, G. Choi, S. Kim, J. Kim, M. Kang, H.E. Jeong, 3D Printable Self-Adhesive and Self-Healing Ionotronic Hydrogels for Wearable Healthcare Devices, ACS Appl. Mater. Interfaces 15 (2023) 11042-11052. <https://doi.org/10.1021/acsami.2c21704>.

[10] M. Suneetha, K.M. Rao, S.S. Han, Mussel-Inspired Cell/Tissue-Adhesive, Hemostatic Hydrogels for Tissue Engineering Applications, ACS Omega 4 (2019) 12647-12656. <https://doi.org/10.1021/acsomega.9b01302>.

[11] J. Deng, H. Yuk, J.J. Wu, C.E. Varela, X.Y. Chen, E.T. Roche, C.F. Guo, X.H. Zhao, Electrical bioadhesive interface for bioelectronics, Nat. Mater. 20 (2021) 229-236. <https://doi.org/10.1038/s41563-020-00814-2>.

[12] M. Sun, P. Li, H. Qin, N. Liu, H. Ma, Z. Zhang, J. Li, B. Lu, X. Pan, L. Wu, Liquid metal/CNTs hydrogel-based transparent strain sensor for wireless health monitoring of aquatic animals, Chem. Eng. J. 454 (2023). <https://doi.org/10.1016/j.cej.2022.140459>.

[13] H. Zhou, J. Lai, B. Zheng, X. Jin, G. Zhao, H. Liu, W. Chen, A. Ma, X. Li, Y. Wu, From Glutinous-Rice-Inspired Adhesive Organohydrogels to Flexible Electronic Devices Toward Wearable Sensing, Power Supply, and Energy Storage, Adv. Funct. Mater. 32 (2022) 2108423. <https://doi.org/10.1002/adfm.202108423>.

[14] H. Yuk, C.E. Varela, C.S. Nabzdyk, X.Y. Mao, R.F. Padera, E.T. Roche, X.H. Zhao, Dry double-sided tape for adhesion of wet tissues and devices, Nature 575 (2019) 169. <https://doi.org/10.1038/s41586-019-1710-5>.
